# Matched-pair analysis: identification of factors with independent influence on the development of PTLD after kidney or liver transplantation

**DOI:** 10.1186/s13737-016-0036-1

**Published:** 2016-08-02

**Authors:** Lisa Rausch, Christian Koenecke, Hans-Friedrich Koch, Alexander Kaltenborn, Nikos Emmanouilidis, Lars Pape, Frank Lehner, Viktor Arelin, Ulrich Baumann, Harald Schrem

**Affiliations:** 1 Core Facility Quality Management & Health Technology Assessment in Transplantation, Integrated Research and Treatment Center Transplantation (IFB-Tx), Hannover Medical School, Hannover, Germany; 2 Hematology, Hemostasis, Oncology and Stem Cell Transplantation, Hannover Medical School, Hannover, Germany; 3 Trauma and Orthopedic Surgery, Federal Armed Forces Hospital Westerstede, Westerstede, Germany; 4 General, Visceral and Transplant Surgery, Hannover Medical School, Carl-Neuberg-Str. 1, 30625 Hannover, Germany; 5 Pediatric Nephrology, Hepatology and Metabolic Disorders, Hannover Medical School, Hannover, Germany; 6 Nephrology, Hannover Medical School, Hannover, Germany

**Keywords:** Mortality, Immunosuppression, Tacrolimus, CMV infection, Human leukocyte antigen

## Abstract

**Background:**

Post-transplant lymphoproliferative disorder (PTLD) adversely affects patients’ long-term outcome.

**Methods:**

The paired *t* test and McNemar’s test were applied in a retrospective 1:1 matched-pair analysis including 36 patients with PTLD and 36 patients without PTLD after kidney or liver transplantation. Matching criteria were age, gender, indication, type of transplantation, and duration of follow-up. All investigated PTLD specimen were histologically positive for EBV. Risk-adjusted multivariable regression analysis was used to identify independence of risk factors for PTLD detected in matched-pair analysis. The resultant prognostic model was assessed with ROC-curve analysis.

**Results:**

Patients suffering with PTLD had shorter mean survival (*p* = 0.004), more episodes of CMV infections or reactivations (*p* = 0.042), and fewer recipient HLA A2 haplotypes (*p* = 0.007), a tacrolimus-based immunosuppressive regimen (*p* = 0.052) and higher dosages of tacrolimus at hospital discharge (Tac dosage) (*p* = 0.052). Significant independent risk factors for PTLD were recipient HLA A2 (OR = 0.07, 95 % CI = 0.01–0.55, *p* = 0.011), higher Tac dosages (OR = 1.29, 95 % CI = 1.01–1.64, *p* = 0.040), and higher numbers of graft rejection episodes (OR = 0.38, 95 % CI = 0.17–0.87, *p* = 0.023). The following prognostic model for the prediction of PTLD demonstrated good model fit and a large area under the ROC curve (0.823): PTLD probability in % = Exp(*y*)/(1 + Exp(*y*)) with *y* = 0.671 − 1.096 × HLA A2-positive recipient + 0.151 × Tac dosage − 0.805 × number of graft rejection episodes.

**Conclusions:**

This study suggests prognostic relevance for recipient HLA A2, CMV, and EBV infections or reactivations and strong initial tacrolimus-based immunosuppression. Patients with risk factors may benefit from intensified screening for PTLD.

## Background

Post-transplant lymphoproliferative disorders (PTLD) are a heterogeneous group of diseases [1]. A clear definition is difficult since PTLD include a wide spectrum from lymphoid hyperplasia, like mononucleosis, to atypical lymphoid hyperplasia with beginning effacement of normal tissue architecture, to an infiltrative type of polyclonal to monoclonal lymphoma [2, 3]. Despite these taxonomical imprecisions, PTLD is clearly a serious and life-threatening complication after transplantation with a high mortality rate of 30–60 % [4].

The underlying disease leading to transplantation might have an influence on the risk of developing PTLD [5]. It was reported that patients under the age of 10, as well as patients over 60 years of age, are more likely to develop PTLD [1, 6–8]. Furthermore, pre-transplant malignancy is supposed to increase the risk of PTLD [1, 9, 10]. Human leukocyte antigen (HLA) types HLA-A2, HLA-A11, HLA-B5, HLA-B18, HLA-B21, and HLA-B35 in transplant recipients as well as HLA-B40 group in EBV-seropositive and HLA-B8 in EBV-seronegative patients were described to be associated with an increased risk for PTLD, whereas HLA-A3 and HLA-DR7 appear to decrease the risk [11–13]. Beside the recipients’ HLA status, the donors’ HLA typing result as well as the matching or mismatching of both seems to have an influence on the development of PTLD [1, 4, 14–16].

There is some evidence that the type and intensity of immunosuppression influences the risk of PTLD [7–9, 17–23].

The majority of PTLDs are associated with EBV infection [1, 24–29], and thus the risk for PTLD depends on the donor’s and recipient’s EBV status [1, 24, 29, 30]. In conjunction with this observation, it has been described that antiviral agents, such as ganciclovir, aciclovir, and foscarnet, may be useful for therapy and prophylaxis of EBV-related PTLD [1, 31–34].

Coexisting CMV infection might be a risk factor for PTLD [7, 35, 36]. However, this association has not been confirmed yet [5, 37, 38]. Infections with the hepatitis C virus and human herpes virus 8 have also been described as risk factors for PTLD [1, 36, 39, 40].

This study aims to evaluate the relevance of risk factors for PTLD development in adult and pediatric patients after primary kidney or liver transplantation independent of age, gender, indication for transplantation, type of transplantation, year of transplantation, and duration of follow-up by using these factors as matching criteria for a matched-pair analysis.

## Methods

### Setting

A university hospital in Germany provides the setting within the Eurotransplant community.

### Study type and study population

This is a single-center retrospective observational 1:1 matched-pair analysis based on 2897 patients who underwent primary liver transplantation between 01.01.1983 and 31.12.2012, as well as 1895 patients who underwent primary kidney transplantation between 01.01.2000 and 31.12.2012 at Hannover Medical School. Combined transplants were excluded due to a lack of adequate matching partners.

### Definition of matching criteria

Each analyzed case with PTLD during follow-up was matched with one non-PTLD case applying the matching criteria age at transplantation, gender, indication for transplantation, type of transplantation, year of transplantation, and duration of post-transplant follow-up. In non-PTLD patients, the duration of follow-up for matching was defined as time to PTLD diagnosis of the matching partner.

### Definition of analyzed variables

Concerning donor and recipient HLA status, we only considered the donors’ HLA of the actual transplanted organ at PTLD diagnosis, which may be relevant in cases with multiple transplants during follow-up (*n* = 5). The same principle was applied to the matching partners without PTLD with subsequent transplants during follow-up (*n* = 6): the donors’ HLA at the equivalent time point to the diagnosis of PTLD in the corresponding partner was considered.

Borderline changes in the transplanted organ were categorized according to the Banff classification and classified as graft rejections for the purpose of this study, because these changes were typically treated with high dose steroids, just as in acute graft rejection which is common practice in many centers [41].

In the absence of an explicit proof of infection, children under the age of 1 year were considered seronegative for viral infections as maternal antibodies may give false positive antibody status [21].

Quantitative data on immunosuppressive therapy of our patients was collected from hospital discharge after transplantation until the time of PTLD diagnosis or equivalent time periods for the non-PTLD matching partners. The duration of given immunosuppressive drugs in months and all changes of the type of immunosuppression were analyzed, and percentages of the follow-up time with either ciclosporine-based or tacrolimus-based immunosuppression were calculated. If a patient was not receiving tacrolimus, ciclosporine, etc., the value was set to zero.

### Statistical methods

**Fig. 1:**
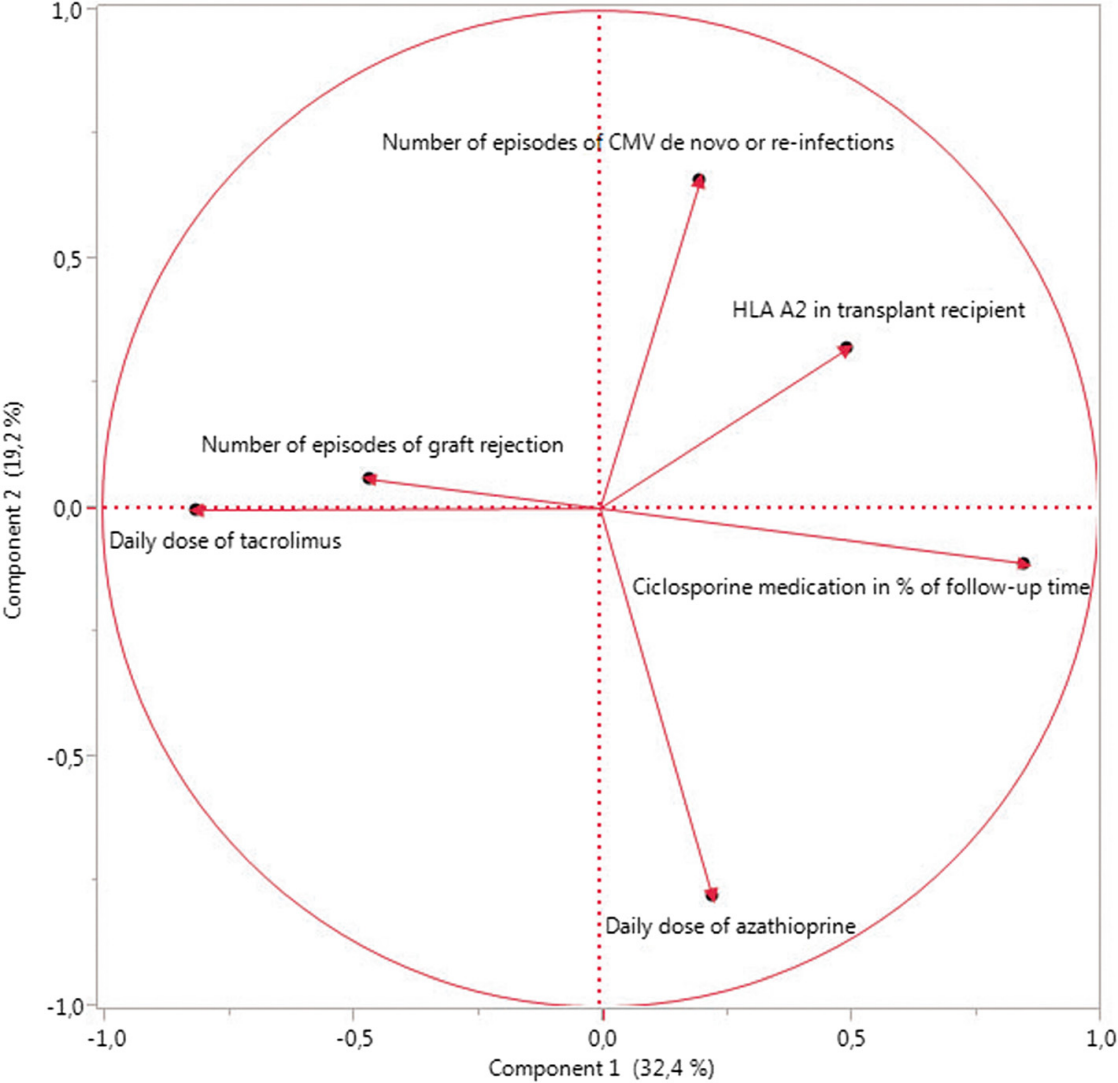
Shown is the result of principal component analysis of analyzed risk factors prior to inclusion into multivariable regression models. This result demonstrates lack of relevant multicollinearity of these factors

In matched-pair analysis, all continuous variables were analyzed using the paired *t* test while binominal variables were analyzed using McNemar’s test. Multivariable principal component analysis was used to understand the underlying data structure and to define uncorrelated variables for prognostic score design by avoiding multicollinearity in regression (Fig. 1). After exclusion of multicollinearity, all variables with an alpha-error <0.200 in univariable analyses (paired *t* test for continuous variables and McNemar’s test for binominal variables) were included into multivariable conditional logistic regression analysis for binary matched pairs as described by Agresti [42] with the goal to develop a regression model for the prediction of PTLD and to identify significant risk-adjusted independent risk factors for PTLD. Model fit was assessed for regression models using Hosmer-Lemeshow’s chi-square test. ROC-curve analysis was performed to calculate the sensitivity and specificity of the derived regression model for the prediction of PTLD [43, 44]. Determination of the area under the ROC-curve (AUROC) was used to assess the potential clinical usefulness of the final prediction model [43, 44] derived with the logit link function.

All *p* values <0.05 were defined as significant. Analyses were performed using JMP® software, version 11, from SAS Institute Inc., Cary, NC, USA.

## Results

### Identification of PTLD cases and their inclusion into matched-pair analysis

A total of 41 cases with PTLD were identified. Thirty-six of these 41 patients with PTLD were included into the matched-pair analysis, with 16 of them being primary kidney-transplanted patients and 20 primary liver transplanted patients. Five patients with PTLD were excluded due to the lack of available data on important risk factors or inability to find an appropriate matching case without PTLD during follow-up.

Within the group of 16 kidney-transplanted patients, nine were female and seven were male with nine being transplanted as a child and seven as an adult. The median age at the transplantation of kidney-transplanted patients with PTLD was 14.4 years (mean age 23.6 years) whereas the median age of their non-PTLD counterparts was 15.6 years (mean age 26.6). All of them were transplanted between 2000 and 2010. The underlying diseases leading to transplantation form a heterogeneous group of diseases, varying between different types of glomerulonephritis to kidney dysplasia, autosomal dominant polycystic disease, hemolytic-uremic syndrome, and others.

Within the group of 20 liver-transplanted patients, five were female and 15 were male with 12 of them being transplanted as a child and eight being transplanted as an adult. The median age at transplantation in the PTLD group was 5.5 years (mean age 21.2 years) as compared to 7.8 years (mean age 21.3 years) in the non-PTLD group. Here, the underlying diseases leading to transplantation were biliary atresia in many cases and less frequently primary sclerosing cholangitis, viral-related cirrhosis and acute liver failure.

The mean differences of the date of transplantation and the age at transplantation between matches were 0.02 days (standard error 103.8 days, *p* = 0.147; paired *t* test) and 0.04 years (standard error 0.33 years, *p* = 0.901; paired *t* test), respectively. All other matching criteria were 100 % identical matches.

### Clinicopathological characteristics of analyzed PTLD cases

Within 36 analyzed PTLD cases, 16 underwent kidney transplantation and 20 underwent liver transplantation. Twenty-one of 36 patients were transplanted as children (age <17 years) and 15 as adults, with 19 of them being diagnosed for PTLD during childhood (8 kidney-transplanted cases and 11 liver-transplanted cases) versus 17 adult PLTD patients. The mean time to PTLD diagnosis was 3.8 years, and the median age of the patients at PTLD diagnosis was 15.7 years (range 0.8–70.8 years, mean 26.1 years).

Overall mortality during follow-up was 36.1 % in PTLD patients with a mean survival of 294 days after PTLD diagnosis (median 119 days, range 11–953 days). Eleven of the 13 patients who died after PTLD diagnosis had a late PTLD (>365 days after transplantation) and one had a very early PTLD (<183 days after transplantation). The mean age at PTLD diagnosis of these 13 PTLD cases that subsequently died was 32.7 years (median 43.4 years, range 2.2–63.8 years). All of them died after 01.01.2000, and 5 of these patients died after 01.01.2010. Twelve of these 13 patients had a monomorphic PTLD while 8 of them had PTLD disease classified as diffuse large B-cell lymphoma. Ten of the deceased patients had extranodal disease with five patients suffering from CNS involvement and three patients having a primary CNS lymphoma. Six of these 13 patients were treated with rituximab with or without chemotherapy and five patients were treated with radiotherapy. In six of 11 patients who subsequently died, immunosuppressive therapy was changed after PTLD diagnosis while in five of 11 patients who died received antiviral therapy.

The distributions of the clinicopathological characteristics of 36 analyzed PTLD cases are summarized in Table 1.

**Table 1:** Clinicopathological characteristics of all PTLD patients

Variable	Distribution
Age at transplantation in years	Median: 12.7 (0.4–65.4); mean: 22.3
Time to diagnosis after transplantation in years	Median: 2.1 (0.3–19); mean: 3.8
Age at PTLD diagnosis in years	Median: 15.7 (0.8–70.8); mean: 26.1
Age > 60 years at PTLD diagnosis	*n* = 4 (11.1 %)
Age < 10 years at PTLD diagnosis	*n* = 14 (38.9 %)
Age < 5 years at PTLD diagnosis	*n* = 11 (30.6 %)
Late PTLD (>365 days after Tx)	*n* = 24 (66.7 %)
Very early PLTD (<183 days after Tx)	*n* = 4 (11.1 %)
Ciclosporine at PTLD diagnosis	*n* = 15 (41.7 %)
Tacrolimus at PTLD diagnosis	*n* = 16 (44.4 %)
CNI-free immunosuppression at PTLD diagnosis	*n* = 5 (13.9 %)
Mycophenolat at PTLD diagnosis	*n* = 19 (52.8 %)
Steroids at PTLD diagnosis	*n* = 29 (80.6 %)
Steroid-free immunosuppression at PTLD diagnosis	*n* = 7 (19.4 %)
mTOR inhibitors at PTLD diagnosis	*n* = 1 (2.8 %)
Number of graft rejections prior to PTLD diagnosis	Median: 0 (0–2); mean: 0.44
Number of graft rejections after PTLD diagnosis	Median: 0 (0–1); mean: 0.25
Polymorphic PTLD	*n* = 5 (13.9 %)
Monomorphic PTLD	*n* = 28 (77.8 %)
Pure B cell neoplasm	*n* = 29 (80.6 %)
Diffuse large B cell neoplasm	*n* = 20 (55.6 %)
CD20 expression in tumor	*n* = 32 (88.9 %)
EBV latent membrane protein or EBV-encoded RNA in tumor cells	*n* = 26 (100.0 %^a^, 10 cases missing data)
Detection of monoclonal disease	*n* = 9 (52.9 %^a^, 19 cases missing data)
Extranodal disease	*n* = 24 (66.7 %)
Graft organ involvement	*n* = 4 (11.1 %)
CNS involvement	*n* = 7 (19.4 %)
Primary CNS lymphoma	*n* = 4 (11.1 %)
Bone marrow involvement	*n* = 6 (16.7 %)
Gastro-intestinal involvement	*n* = 14 (38.9 %)
Lung involvement	*n* = 3 (8.3 %)
Skin involvement	*n* = 0 (0.0 %)
Number of sites involved	Median: 2 (1–6); mean: 2.5
Stage IV disease	*n* = 25 (69.4 %)
B-symptoms at PTLD diagnosis	*n* = 4 (11.1 %)
Lactate dehydrogenase elevated at PTLD diagnosis	*n* = 23 (71.9 %^a^, 4 cases missing data)
Hypoalbuminemia at PTLD diagnosis	*n* = 17 (65.4 %^a^, 10 cases missing data)
EBV IgG at Tx	*n* = 14 (45.2 %^a^, 5 cases missing data)
EBV IgM at Tx	*n* = 1 (3.3 %^a^, 6 cases missing data)
EBV serology or DNA positive at PTLD diagnosis	*n* = 31 (100.0 %^a^, 5 cases missing data)
EBV DNA at PTLD diagnosis	*n* = 29 (96.7 %^a^, 6 cases missing data)
EBV IgG at PTLD diagnosis	*n* = 21 (77.8 %^a^, 9 cases missing data)
CMV IgG at Tx	*n* = 12 (35.3 %^a^, 2 cases missing data)
CMV IgM at Tx	*n* = 3 (9.7 %^a^, 5 cases missing data)
CMV pp65 at Tx	*n* = 1 (3.8 %^a^, 10 cases missing data)
CMV serology or DNA positive at PTLD diagnosis	*n* = 23 (100 %^a^, 13 cases missing data)
CMV pp65 at PTLD diagnosis	*n* = 1 (4.2 %^a^, 12 cases missing data)
CMV DNA at PTLD diagnosis	*n* = 4 (22.2 %^a^, 18 cases missing data)
CMV IgM at PTLD diagnosis	*n* = 7 (30.4 %^a^, 13 cases missing data)
CMV IgG at PTLD diagnosis	*n* = 21 (77.8 %^a^, 9 cases missing data)

Shown are the distributions of clinicopathological characteristics of 36 analyzed PTLD cases

*Tx* transplantation, *CNI-free* calcineurin-inhibitor-free, *EBV* Epstein-Barr virus, *CMV* cytomegalovirus

^a^Of evaluated cases

### Survival in PTLD versus matched non-PTLD patients

**Fig. 2:**
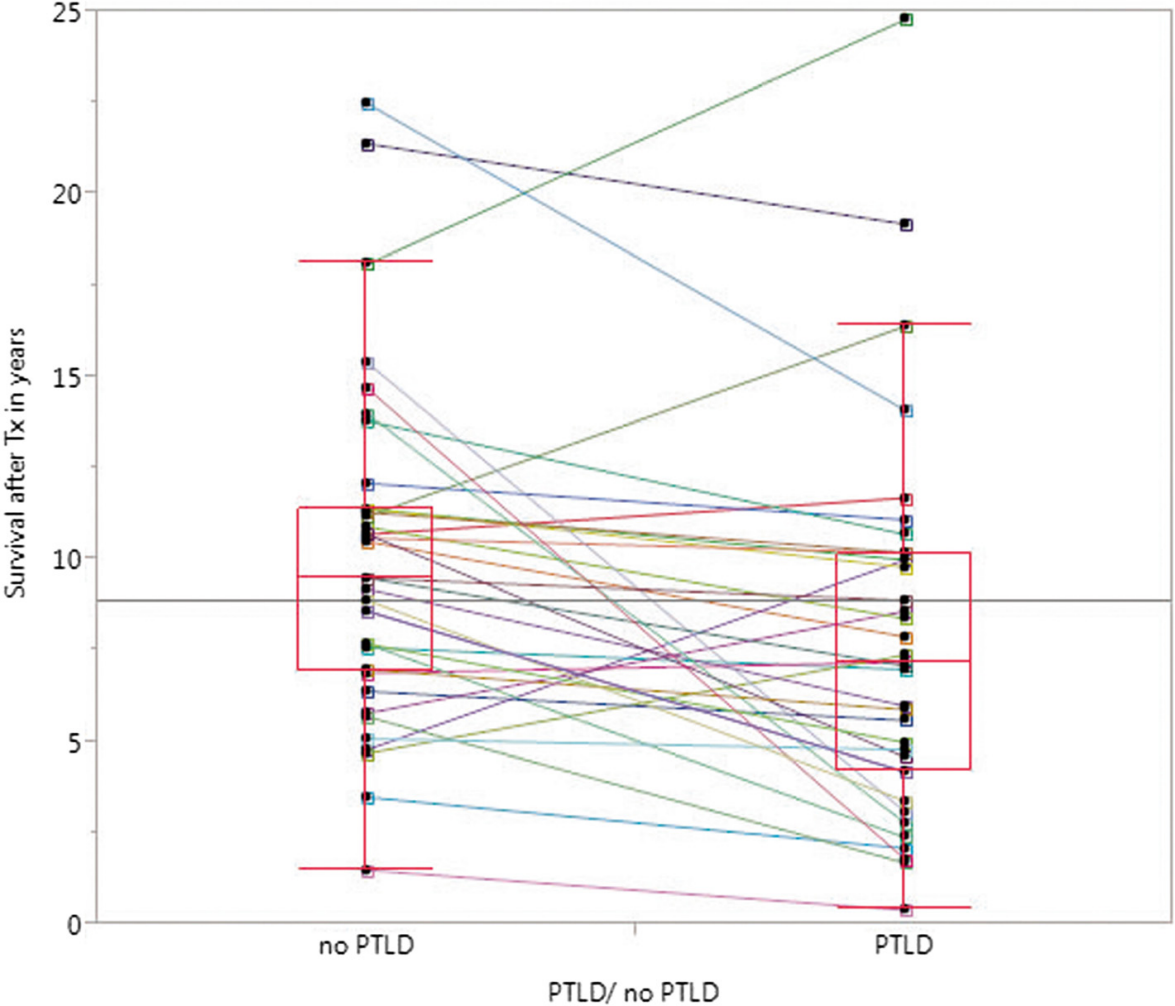
Shown are the results of matched-pair analysis for patient survival after transplantation (*p* = 0.004; paired *t* test). The *colored lines* link values between PTLD patients and matching non-PTLD partners, demonstrating the difference in each pair. Tx transplantation

Only in one pair the non-PTLD patient died within the timeframe in which the PTLD-match was still alive. In contrast, 11 PTLD patients died during follow-up while their non-PTLD matching partners continued to survive (*p* = 0.004, McNemar’s test) (Table 2). Mean survival after the transplantation of PTLD patients (7.74 years) was significantly shorter as compared to the matched non-PTLD partners (9.98 years) (*p* = 0.004, paired *t* test) (Table 3, Fig. 2).

**Table 2:** Matched-pair analysis for binominal variables

Variables (+)/(−)^a^	Nr. of analyzed matched pairs in %	Concordant pairs		Discordant pairs		*p* value^b^
		Combination PTLD (+) and non-PTLD (+)	Combination PTLD (−) and non-PTLD (−)	Combination PTLD (−) and non-PTLD (+)	Combination PTLD (+) and non-PTLD (−)	
Death	100.0	2	22	*1*	*11*	*0.004*
Subsequent re-transplants	100.0	1	26	5	4	0.739
Living Donor	100.0	4	18	5	9	0.285
BMI >30 kg/m^2^	100.0	0	32	2	1	0.564
Diabetes	100.0	0	32	2	2	1.000
Alcohol abuse	100.0	0	34	2	0	n.d.
Active Smoking	100.0	0	31	2	3	0.655
COPD	100.0	0	35	1	0	n.d.
Recipient EBV IgG at Tx	86.1	12	13	4	2	0.414
Recipient CMV IgG at Tx	91.7	9	15	6	3	0.317
Donor CMV IgG at Tx	97.2	13	8	5	9	0.285
Recipient Anti-HCV at Tx	61.1	0	22	0	0	n.d.
Anti-CMV treatment after Tx	100.0	10	9	7	10	0.467
Ganciclovir/valganciclovir after Tx	100.0	9	11	7	9	0.617
Pre-transplant malignancy	100.0	2	34	0	0	1.000
Pre-transplant HCC	100.0	2	34	0	0	1.000
Non-PTLD malignancy after Tx	100.0	0	31	2	3	0.655
Breast cancer after Tx	100.0	0	35	0	1	n.d.
Pre-transplant dialysis	100.0	12	20	2	2	1.000
Donor HLA A26, B38	75.0	0	25	0	2	n.d.
Donor HLA A1	75.0	1	15	5	6	0.763
Donor HLA B8	75.0	1	21	3	2	0.655
Donor HLA DR3	75.0	1	20	4	2	0.414
Recipient HLA A26, B38	52.8	0	18	0	1	n.d.
Recipient HLA A2	52.8	6	2	*10*	*1*	*0.007*
Recipient HLA A11	52.8	0	15	3	1	0.317
Recipient HLA B5	52.8	0	15	2	2	1.000
Recipient HLA B18	52.8	0	16	3	0	n.d.
Recipient HLA B21	52.8	0	18	1	0	n.d.
Recipient HLA B35	52.8	1	12	4	2	0.414
Recipient HLA A3	52.8	1	10	4	4	1.000
Recipient HLA DR27	52.8	0	19	0	0	n.d.
HLA A locus mismatching	47.2	7	2	3	5	0.480
HLA B locus mismatching	47.2	10	0	3	4	0.706
HLA DR locus mismatching	47.2	12	2	2	1	0.564
Anti-thymocyte globulin after Tx	86.1	0	30	1	0	n.d.
Basiliximab therapy after Tx	86.1	6	11	7	7	1.000
Daclizumab therapy after Tx	86.1	0	29	1	1	1.000
Tacrolimus^c^ after Tx	100.0	5	18	*3*	*10*	*0.052*
Ciclosporine^c^ after Tx	100.0	17	5	10	4	0.109
MMF^c^ after Tx	100.0	10	15	5	6	0.763
Myfortic^c^ after Tx	100.0	0	35	0	1	n.d.
Steroids^c^ after Tx	100.0	32	1	1	2	0.564
Sirolimus^c^ after Tx	100.0	0	34	1	1	1.000
Everolimus^c^ after Tx	100.0	0	34	1	1	1.000
Azathioprin^c^ after Tx	100.0	1	33	2	0	0.157
Tacrolimus at PTLD^d^	100.0	7	14	6	9	0.439
Ciclosporine at PTLD^d^	100.0	12	12	9	3	0.083
CNI-free treatment at PTLD^d^	100.0	1	30	1	4	0.180

Summarized are the results of matched-pair analysis for binominal variables. Their distribution in concordant and discordant pairs in matched-pair analysis is shown

^a^(+)/(−)-classifiers identify yes/no or positive/negative variables

^b^McNemar’s test for binary variables

^c^At hospital discharge

^d^Diagnosis or equivalent date in the non-PTLD group

*Tx* transplantation, *COPD* chronic obstructive pulmonary disease, *BMI* body mass index, *EBV* Epstein-Barr virus, *CMV* cytomegalovirus, *HCC* hepatocellular carcinoma, *HCV* hepatits C virus, *HLA* human leukocyte antigen, *MMF* mycophenolat mofetil, *CNI* calcineurin inhibitor, *n.d. p* value not determined due to zero cases within pairs (McNemar’s test)

**Table 3:** Matched-pair analysis for continuous variables

Variable	PTLD mean	Non-PTLD mean	Mean difference of Non-PTLD minus PTLD	*p* value^a^	Standard error of mean difference
Survival after Tx in years	7.74	9.98	2.24	*0.004*	0.72
PTLD-free survival in years	3.99	9.98	5.99	*<0.001*	0.76
Number of subsequent re-transplants	0.17	0.17	0.00	1.000	0.10
CIT in minutes	658.07	627.07	−31.00	0.756	99.05
BMI in kg/m^2^	20.05	20.13	0.09	0.890	0.62
Number of episodes of graft rejection	0.71	1.17	0.46	0.118	0.29
Number of episodes of EBV de novo infections or reactivations	0.50	0.50	0.00	1.000	0.15
Number of episodes of CMV de novo infections or reactivations	1.47	0.75	−0.72	*0.042*	0.34
Duration of pre-Tx dialysis in years	4.02	4.65	0.63	0.561	1.06
Number of HLA A locus recipient-donor mismatches	0.76	0.71	−0.06	0.791	0.22
Number of HLA B locus recipient-donor mismatches	1.12	1.06	−0.06	0.805	0.23
Number of HLA DR locus recipient-donor mismatches	0.94	1.06	0.12	0.431	0.15
Overall number of HLA recipient-donor mismatches	2.82	2.82	0.00	1.000	0.51
Daily dose of tacrolimus in mg^b^ after Tx	3.74	1.62	−2.12	*0.052*	1.05
Blood level of tacrolimus in ng/ml^b^ after Tx	9.97	8.13	−1.83	0.734	4.69
Daily dose of ciclosporine in mg^b^ after Tx	176.17	183.06	6.89	0.832	32.24
C0 blood level of ciclosporine in μg/l^b^ after Tx	218.46	195.91	−22.55	0.230	17.63
C2 blood level of ciclosporine in μg/l^b^ after Tx	1183.0	1168.5	−14.5	0.909	100.5
Daily dose of MMF in mg after^b^ after Tx	545.00	551.67	6.67	0.967	161.43
Daily dose of prednisolone in mg^b^ after Tx	11.13	10.99	−0.14	0.916	1.31
Daily dose of sirolimus in^b^ after Tx	0.06	0.06	0.00	1.000	0.08
Daily dose of everolimus in mg^b^ after Tx	0.06	0.14	0.08	0.585	0.15
Daily dose of azathioprine in mg^b^ after Tx	2.08	2.78	0.69	0.160	0.48
Tacrolimus treatment in months ^c^	12.47	15.62	3.15	0.596	5.87
Tacrolimus treatment in % of time of immunosuppression^c^	46.87	33.45	−13.42	0.231	11.00
Ciclosporine treatment in months^c^	27.16	25.58	−1.58	0.737	4.67
Ciclosporine treatment in % of time of immunosuppression^c^	46.25	59.70	13.45	0.180	9.83
CNI-free treatment in months^c^	6.96	5.29	−1.67	0.628	3.41
CNI-free treatment in % of time of immunosuppression^c^	7.81	6.69	−1.11	0.803	4.42

Shown are the results of univariable matched-pair analysis for continuous variables

^a^Paired *t* test

^b^At hospital discharge

^c^Until PTLD diagnosis or equivalent date in the non-PTLD group)

*Tx* transplantation, *CIT* cold ischemic time, *BMI* body mass index, *EBV* Epstein-Barr virus, *CMV* cytomegalovirus, *HLA* human leukocyte antigen, *C0* trough blood level, *C2* blood level 2 hours after oral intake, *MMF* mycophenolat mofetil, *CNI* calcineurin inhibitor

### Significant risk factors for PTLD development in matched-pair analysis

**Fig. 3:**
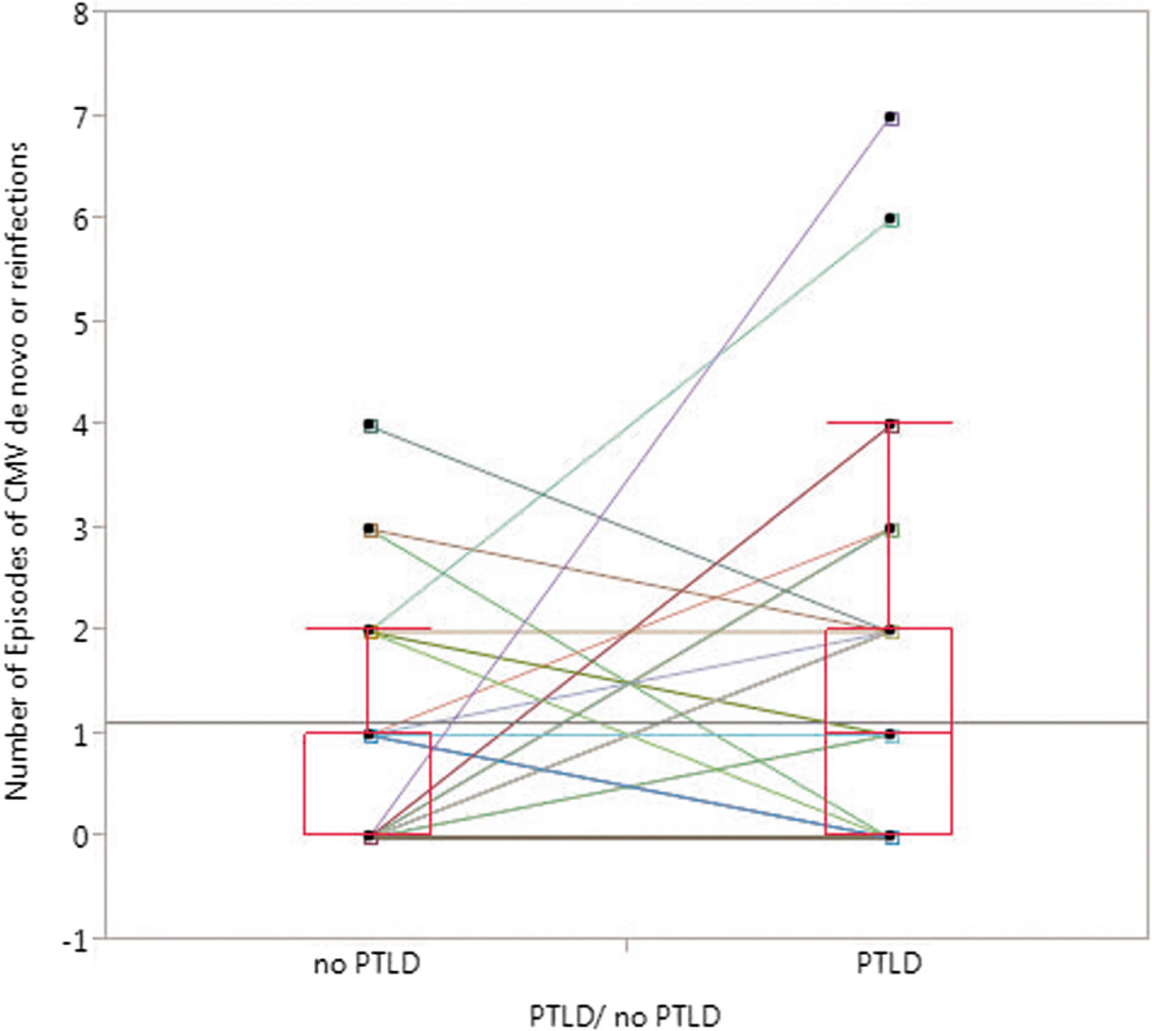
Shown are the results of matched-pair analysis for the number of episodes of CMV de novo infections or reactivations (*p* = 0.042; paired *t* test). The *colored lines* link values between PTLD patients and matching non-PTLD partners, demonstrating the difference in each pair. *Tx* transplantation, *CMV* cytomegalovirus

The number of episodes of CMV de novo infections or reactivations showed a significant difference between PTLD cases (mean: 1.47) and their non-PTLD matching partners (mean: 0.75) (*p* = 0.042, paired *t* test) (Table 3, Fig. 3).

The recipients’ HLA A2 was significantly more frequent in the non-PTLD matching partners. In 10 out of 19 analyzed pairs, the PTLD patient was negative for HLA A2 while the matching partners were HLA A2 positive and only in one discordant pair, we found the opposite result (*p* = 0.007, McNemar’s test) (Table 2).

**Fig. 4:**
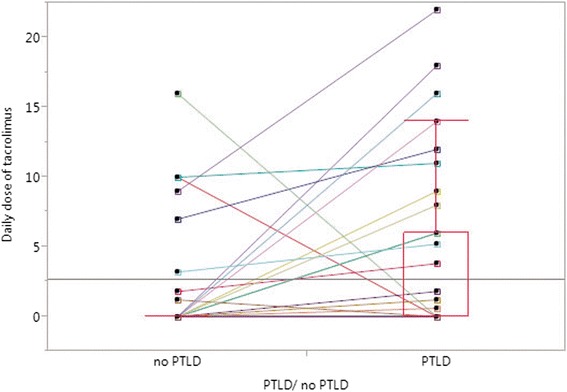
Shown are the results of matched-pair analysis for daily dosages of tacrolimus at hospital discharge after transplantation (*p* = 0.052; paired *t* test). The *colored lines* link values between PTLD patients and matching non-PTLD partners, demonstrating the difference in each pair. *Tx* transplantation

Immunosuppression with tacrolimus at the time of hospital discharge after transplantation was associated with later development of PTLD (*p* = 0.052; McNemar’s test). In ten pairs, the patient with PTLD was treated with tacrolimus at hospital discharge while their respective matching partners received ciclosporine-based immunosuppression instead. In three discordant cases, the opposite was the case (Table 2). The mean daily dose of tacrolimus at hospital discharge was significantly higher within the PTLD group (3.74 mg) as compared to their non-PTLD matching partners (1.62 mg) (*p* = 0.052, paired *t* test) (Table 3, Fig. 4). However, blood levels of tacrolimus or ciclosporine (trough blood level as well as blood level 2 hours after oral intake) at hospital discharge did not show to be significantly different between matched pairs (Table 3). Immunosuppressive induction therapy with anti-thymocyte globulin (ATG), basiliximab, and daclizumab did not differ significantly between PTLD and non-PTLD patients. In matched-pair analysis, an equal number of discordant pairs resolved in a non-significant *p* value for immunosuppressive induction therapies either with basiliximab or daclizumab (Table 2).

All other analyzed risk factors did not show any statistically significant differences between the PTLD cases and their matched controls (Tables 2 and 3). In particular, the recipients’ EBV serology status at transplantation was not significantly different between PTLD cases and their matching partners (*p* > 0.05, McNemar’s test). In four pairs, the PTLD patient was EBV IgG negative at transplantation and the matching non-PTLD partner was EBV IgG positive, whereas in two pairs, the opposite was observed with the PTLD case EBV IgG positive and the matching partner EBV IgG negative at transplantation (*p* = 0.414; McNemar’s test). Analysis of ganciclovir or valganciclovir treatment after transplantation revealed seven matching pairs where the PTLD patient did not receive ganciclovir or valganciclovir when the non-PTLD patient did, whereas in nine matching pairs, this was the opposite (*p* = 0.617; McNemar’s test).

### Independent risk factors for PTLD development in multivariable conditional logistic regression

Multivariable conditional regression analysis revealed that HLA A2 in the transplant recipient (odds ratio (OR) 0.07, 95 % CI 0.01–0.55, *p* = 0.011), the level of tacrolimus dosing at hospital discharge after transplantation (OR 1.29, 95 % CI 1.01–1.64, *p* = 0.040) and the number of graft rejection episodes (OR 0.38, 95 % CI 0.17–0.87, *p* = 0.023) were independent significant risk factors after risk adjustment for the matching criteria and the included variables with *p* values <0.200 in univariable analyses (Tables 2 and 3). Multivariable principal component analysis demonstrated lack of multicollinearity between these variables (Fig. 1).

### Multivariable regression model for the prediction of PTLD in matched-pair analysis

The proposed prognostic model for PTLD probability in this matched-pair analysis has been derived with the logit link function of the multivariable binary regression model as:

$$ \begin{array}{c}\mathrm{PTLD}\ \mathrm{p}\mathrm{robability}\ \mathrm{in}\ \% = \mathrm{E}\mathrm{x}\mathrm{p}(y)\ /\ \left(1 + \mathrm{E}\mathrm{x}\mathrm{p}(y)\right)\ \mathrm{with}\hfill \\ {}\begin{array}{l}Y = 0.671 - 1.096 \times \mathrm{H}\mathrm{L}\mathrm{A}\ \mathrm{A}2\ \mathrm{p}\mathrm{ositive}\ \mathrm{recipient} + 0.151 \times \mathrm{tacrolimus}\ \mathrm{dose}\ \mathrm{in}\ \mathrm{mg}\\ {}\ \mathrm{at}\ \mathrm{hospital}\ \mathrm{discharge}\ \mathrm{after}\ \mathrm{transplantation} - 0.805 \times \mathrm{number}\ \mathrm{of}\ \mathrm{episodes}\ \mathrm{of}\\ {}\ \mathrm{graft}\ \mathrm{rejection}\end{array}\hfill \end{array} $$

**Fig. 5:**
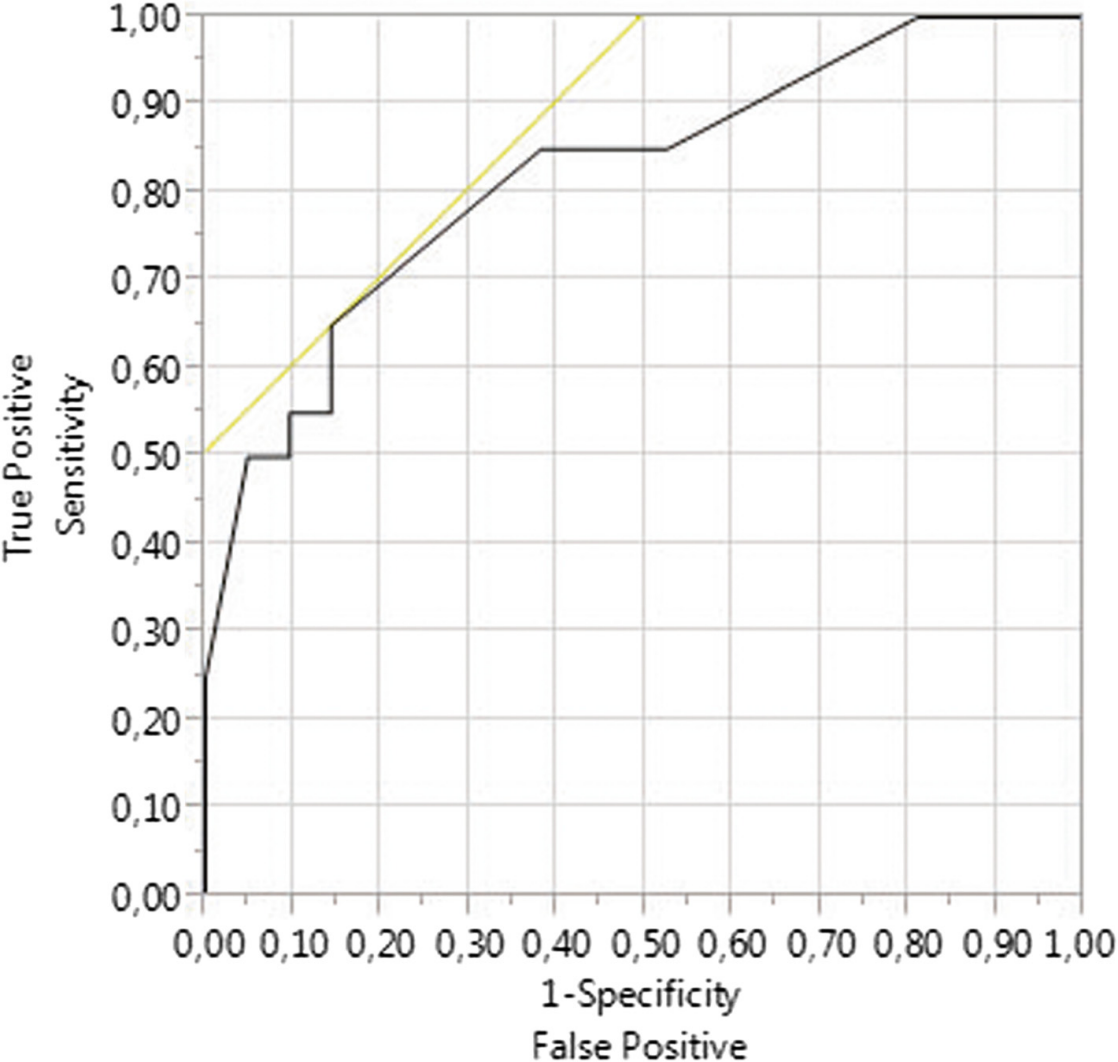
Shown is the result of ROC-curve analysis of the multivariable logistic regression model for the prediction of PTLD in matched-pair analysis (AUROC: 0.823) indicating a good discriminative power as well as good overall model correctness and thus potential clinical usefulness provided that the matched-pair criteria are met

Model fit assessment demonstrated a good model fit (*p* = 0.483). The AUROC was assessed as 0.823 (Fig. 5).

## Discussion

### Advantages of the matched-pair approach

This is the first matched-pair analysis of independent risk factors for PTLD development after kidney or liver transplantation. Matched-pair analysis provides comparatively high levels of evidence for significant findings especially in discordant pairs and in comparatively small sample sizes [45]. The goal of this study is to identify those patients who are at increased risk under comparable clinical conditions in order to provide a rationale for individualized prophylactic measures, intensified screening schemes, and patient information.

The role of risk factors for PTLD was studied independently of age, gender, indication for transplantation, type of transplantation, year of transplantation, and duration of follow-up by using these variables as matching criteria because all of these factors cannot be changed by the treating physician during follow-up. This purposefully chosen matching approach enables the identification of risk factors for PTLD that are independent of these criteria. Some of the identified independent risk factors such as the type and intensity of immunosuppression are under direct control of the treating physician and can thus be altered while other risk factors could be used to decide on increased screening for PTLD.

The setting as 1:1 nearest neighbor matching as compared to a larger matched control is justified due to the fact that poor matches are avoided and an often minimal reduction in power of the statistical analysis is possible [46].

Some of the matching criteria have been described before to influence the risk for PTLD. Patients under the age 10, as well as patients over 60 years, are more likely to develop PTLD [1, 6–8]. PTLD incidence was reported to be lowest after renal transplantation (1–2 %) and moderate after liver transplantation (3–12 %), compared to much higher observed incidences after intestinal or multiorgan transplantation [47]. Specific indications for liver transplantation such as autoimmune hepatitis, primary biliary cirrhosis, alcoholic cirrhosis, and acute liver failure appear to relate to the risk of PTLD development [5].

This study used the duration of follow-up for matching. This additional matching criterion is essential for the detection of risk factors that are independent of the duration of immunosuppression which is thought to be a critical risk factor for the development of PTLD [7–9, 17–23]. A very high percentage of patients does perform their follow-up in our transplant center, as there is a defined program for monitoring each patient and their degree of immunosuppression, especially those patients who develop complications are monitored more closely. Without being able to give exact numbers, loss of follow-up is in our opinion not the reason for a rather low PTLD incidence within our center. PLTD incidence in our cohort was 0.86 % which is in line with other reports suggesting a PTLD incidence after liver or kidney transplantation between 1 and 5 % [48].

The occurrence of subsequent re-transplants and the number of subsequent transplants were not significantly different between matched PTLD and non-PTLD cases (*p* = 0.739, McNemar’s test and *p* = 1.000, paired *t* test, respectively). Therefore, the above described consideration of donor and recipient HLA status at PTLD diagnosis or the equivalent time point during follow-up in their corresponding matching partner appears justified.

### Worse survival of patients with PTLD

Long-term survival after transplantation was significantly shorter in the analyzed PTLD patients as compared to the non-PTLD matching partners (*p* = 0.004). This finding confirms the clinical relevance of PTLD for long-term prognosis after transplantation which is known to be a serious and life-threatening complication after solid-organ transplantation [4].

### CMV and EBV are risk factors for PTLD

This matched-pair study revealed that patients with PTLD had significantly more episodes of cytomegalovirus (CMV) de novo infections or reactivations as compared to their non-PTLD matching partners. This result corresponds to other studies that found coexisting CMV infection as a potential risk factor for PTLD [7, 35, 36]. Immunosuppressive therapy promotes a low control of CMV which has been shown to interfere with the immune system in vivo and in vitro [36, 49, 50]. Also, a strong association between CMV reactivation and EBV reactivation has been demonstrated in a study from Zallio et al. [51]. Thus, closer monitoring for CMV activity as well as the role of antiviral prophylaxis after transplantation should not be underestimated. This approach should be considered even in CMV IgG-positive patients as they may reduce the PTLD incidence after transplantation.

Treatment after transplantation with antiviral agents, such as ganciclovir, valganciclovir, or aciclovir, for which prevention of EBV-related PTLD development has been suggested [1, 31, 34], did not show a statistically significant effect on the occurrence of PTLD in this study. We conclude that antiviral prophylaxis may be ineffective for the prevention of PTLD or, alternatively, was not consistently enough or not long enough applied in the investigated cohort to enable a significant protective effect in PTLD cases. Ganciclovir and valganciclovir are nucleoside analogues that inhibit EBV DNA replication. In cells that are latently infected with EBV and cells of EBV-driven lymphomas, these antiviral agents appear to be ineffective due to a lack of an activating thymidine kinase [47].

EBV-seronegative patients were reported in a non-matched analysis to have a 4.7-fold higher hazard ratio for PTLD as compared to EBV-seropositive recipients due to increased EBV infection risks [30]. However, in the current analysis, the frequency of the recipients’ positive EBV IgG status at transplantation was not significantly different between matching PTLD and non-PTLD partners (Table 2).

It is striking that this first matched-pair analysis did not find a significant risk for PTLD that is associated with the occurrence and number of EBV infections or reactivations after transplantation during follow-up independent of age at transplantation.

However, all analyzed PTLD cases with available data on EBV latent membrane protein or EBV-encoded RNA in tumor cells (26 cases of 36 cases) were positive for EBV in the histology specimen (Table 1). All 31 PTLD cases with available data on EBV IgG positivity or EBV DNA positivity at the time of PTLD diagnosis were either EBV IgG or EBV DNA positive at the time of PTLD diagnosis (Table 1).

These findings support the previously described strong risk for PTLD which is associated with EBV infections and reactivations even though the current matched-pair analysis did not reveal a significant statistical difference in the number of EBV infections or reactivation episodes after transplantation between PTLD and matched non-PTLD cases (Table 3). We believe that this somehow contradictory observation may be due to the fact that EBV infections and reactivations were frequently not detected during follow-up due to subclinical symptoms. This assumption supports the recommendation that EBV serology and EBV viral load should be monitored on a regular basis [52].

### Type of immunosuppression is relevant for PTLD risk

Many authors noted that the type of immunosuppression, as well the degree of immunosuppression influences the risk of PTLD [7–9, 17–23]. This matched-pair analysis confirms that initial tacrolimus-based immunosuppression after transplantation as well as high daily doses of tacrolimus after transplantation both increased the risk of later PTLD development. This corresponds to other reports that described a two- to fivefold increase of PTLD risk with tacrolimus instead of ciclosporine after pediatric and adult solid-organ transplantation [8, 53, 54].

Interestingly, immunosuppressive induction therapy, either with basiliximab, daclizumab, or anti-thymocyte globulin (ATG), did not increase or decrease the risk of PTLD development in the setting of this analysis. This finding is based on the fact that no discordant pairs were found where the PTLD case has received induction immunosuppression either with anti-thymocyte globulin, basiliximab, or daclizumab while his or her matching partner did not (Table 2).

The same non-significant result was detected for immunosuppressive blood levels. Neither for tacrolimus nor for ciclosporine blood levels (C0, C2) at hospital discharge, a significant difference between PTLD patients and matched non-PTLD controls could be detected in this study. One explanation could be that none of these blood levels reflect the actual peak drug level which might be the crucial factor for turning cells at risk to dedifferentiate into malignant lymphoma cells.

It is possible that other parameters such as the area under the time curve (AUC) of calcineurin-inhibitor (CNI) blood levels, which is known to be the biologically relevant parameter for toxicity and the immunosuppressive effect of these agents [55, 56], rather than trough blood levels may be more relevant for the development of de novo malignancy. Further, inter-individual differences concerning the first pass metabolism in immunosuppressed patients vary relative to CNI-absorption and thus blood levels [57, 58]. These assumptions may explain to some degree the perceived discrepancy between the observed effects of immunosuppressive dosages on PTLD risk while the respective blood drug levels appeared to be less important.

Further analysis of the duration of treatment with tacrolimus in months as well as in percent of the time until PTLD diagnosis or the equivalent time frame for the non-PTLD matching partners did not reveal a statistically significant difference between PTLD and non-PTLD patients. Similar analyses for the treatment with ciclosporine or calcineurin-inhibitor (CNI)-free immunosuppression during follow-up demonstrated the same non-significant results (Table 3). We believe that analyses of the complete immunosuppression in each individual, including all changes of dosing and all blood level changes over time during follow-up, are required for deeper insights into the long-term consequences of immunosuppression. Dosages in children are usually adapted to their body weight or body surface and blood levels. Nevertheless, we believe that analysis of absolute dosages in combination with blood levels in this study provides reasonable results due to the tight matching for age and duration of follow-up in matched-pair analysis.

The duration of immunosuppression could not be identified as a prognostic variable for PTLD development in this study, because the duration of follow-up after transplantation was used as one of the matching criteria.

### The role of graft rejection and HLA haplotypes

The number of graft rejection episodes appeared to be an independent significant risk factor in multivariable analysis (*p* = 0.023) with higher numbers of graft rejection episodes protecting patients from PTLD (OR = 0.38) which can be explained by lower intensity of immunosuppressive therapy. The likelihood of rejection episodes decreased significantly with higher percentages of time on tacrolimus-based immunosuppression during follow-up (*p* = 0.015, logistic regression) which is in line with previous reports in the literature [59]. This study showed that lower numbers of graft rejection episodes were linked to a higher risk for PTLD, and at the same time, lower numbers of graft rejection episodes were linked to tacrolimus-based immunosuppression reinforcing the finding that tacrolimus dosage at hospital discharge increased the risk of PTLD development. Therefore, patients on tacrolimus with none or few rejection episodes might benefit from closer monitoring for PTLD during follow-up.

HLA A2 haplotype in transplant recipients was a protective factor in this study instead of increasing the risk for PTLD as was reported previously [11, 12]. HLA A2 haplotype encodes a HLA class I protein which presents peptides from expired or defective intracellular proteins as well as proteins from invasive viruses from within the cell to the T cell receptor on CD8+, often cytotoxic, T cells in order to destroy the (infected) cell [60]. The result of our study together with previously published findings suggests that HLA A2 haplotype might be responsible for an effective presentation of EBV antigens to CD8+ cytotoxic T cells and thus that HLA A2-positive individuals may control EBV infection better than negative counterparts [61]. The identification that lack of HLA A2 in the recipient is significantly associated with PTLD is hypothesis generating and should be a basis for larger studies.

Further analysis revealed that the percentage of tacrolimus-based immunosuppression during follow-up and the dosage of tacrolimus at hospital discharge were not related to HLA A2 positivity (*p* = 0.169 and *p* = 0.167, respectively). This finding excludes the possibility that HLA A2 positivity is a surrogate marker for more or less intense immunosuppression.

All other HLA types and possible donor-recipient HLA mismatching combinations which have been previously described as either protective or as risk factors for PTLD development [1, 4, 13–16] did not show any significant influence on PTLD development in this matched-pair analysis. Previous reports did not apply a matched-pair approach. However, this observation is limited by the fact that not for all liver-transplanted patients the HLA status was given and thus taken into account (Table 2).

### Multivariable risk factor analysis

Conditional multivariable regression revealed recipient HLA A2 haplotype, the level of tacrolimus dosage at hospital discharge after transplantation, and the number of graft rejection episodes as independent risk factors for the development of PTLD, provided that the aforementioned matching criteria are met. Addition of the number of CMV infections or reactivations after transplantation as an easily trackable factor to the derived multivariable prediction model did not improve the model while revealing a statistically non-significant influence in multivariable regression. Therefore, we have eliminated this variable from the final prediction model.

The non-significant result of the model fit assessment (*p* = 0.483) excluded the possibility of overfitting, and the AUROC (0.823) of the derived predictive model indicated a good discriminative power as well as good overall model correctness, and thus potential clinical usefulness [43, 44]. It allows an early identification of patients at increased risk for PTLD. We accept that the limitations of the proposed predictive model include the relatively small number of analyzed cases and a lack of external validation with data from another transplant center. The proposed model should therefore be regarded cautiously.

### Limitations

The limitations of this work mostly refer to the limited number of PTLD cases in a single-center study and its retrospective nature. Despite the design of this study as matched-pair analysis and the introduction of several substantial matching criteria, individual differences in patients still contribute to some uncertainty. A larger, prospective, multicenter trial will be necessary to define risk factors for PTLD development and to refine screening methods based on more substantial findings. Caution in the clinical application of the derived prognostic model is warranted due to its current lack of external validation.

## Conclusions

This study shows that the most likely scenario for the development of PTLD is caused by high tacrolimus dosages at hospital discharge, fewer subsequent rejection episodes which are associated with higher percentages of time on tacrolimus treatment during follow-up, absent HLA A2 haplotype in the recipient, and CMV infections or reactivations during follow-up. EBV-related PTLD risk could not be quantified adequately in this study while all investigated tumors were EBV positive. Patients with unfavorable combinations of risk factors should be monitored more closely for PTLD and their EBV status.

## Abbreviations

95 % CI, 95 % confidence interval; ADPKD, autosomal dominant polycystic disease; ATG, anti-thymocyte globulin; AUROC, area under ROC-curve; BMI, body mass index; C0, trough blood level; C2, blood level 2 hours after oral intake; CIT, cold ischemic time; CMV, cytomegalovirus; CNI, calcineurin-inhibitor; CNS, central nervous system; COPD, chronic obstructive pulmonary disease; EBV, Epstein-Barr virus; GN, glomerulonephritis; HCC, hepatocellular carcinoma; HCV, hepatits C virus; HHV-8, human herpes virus 8; HLA, human leukocyte antigen; HUS, hemolytic-uremic syndrome; Ig, immunoglobulin; MMF, mycophenolat mofetil; n.d., not determined; No., number; OR, odds ratio; PSC, primary sclerosing cholangitis; PTLD, post-transplant lymphoproliferative disorder; ROC, receiver operating characteristic; Tx, transplantation
